# Changes in expression of four molecular marker proteins and one microRNA in mesothelial cells of the peritoneal dialysate effluent fluid of peritoneal dialysis patients

**DOI:** 10.3892/etm_2013.1281

**Published:** 2013-09-02

**Authors:** LIN ZHANG, FUYOU LIU, YOUMING PENG, LIN SUN, GUOCHUN CHEN

**Affiliations:** 1 Department of Nephrology, Second Xiangya Hospital, Institute of Nephrology, Central South University, Changsha, Hunan 410011;; 2 Department of Nephrology, Fourth Changsha Hospital, Changsha, Hunan 410006, P.R. China;; 3 Department of Pathology and Medicine, Feinberg School of Medicine, Northwestern University, Chicago, IL 60611, USA

**Keywords:** peritoneal dialysis, peritoneal fibrosis, microRNA-200c

## Abstract

The aim of this study was to detect the expression of microRNA-200c and epithelial-mesenchymal transition (EMT) in the mesothelial cells of the peritoneal dialysate effluent fluid of peritoneal dialysis (PD) patients, and to investigate the association between microRNA-200c and peritoneal mesothelial cell EMT. Twelve patients who had recently started continuous ambulatory peritoneal dialysis (PD start group) and 16 patients who had been undergoing peritoneal dialysis for >6 months (PD >6 months group) were randomly chosen for the isolation, culture and identification of effluent cells. qPCR and western blot analysis were used to detect the expression levels of microRNA-200c and the levels of four cellular marker proteins, E-cadherin, vimentin, fibronectin (FN) and COL-1, in effluent cells. The results showed that the effluent cells in peritoneal dialysis were peritoneal mesothelial cells. The level of E-cadherin protein expression was significantly lower in the PD >6 months group than in the PD start group, while vimentin, FN and COL-1 protein expression levels were significantly increased in the PD >6 months group. microRNA-200c in the PD >6 months group was significantly downregulated. The E-cadherin protein expression level was significantly decreased and vimentin, FN and COL-1 protein expression levels were significantly increased in the PD >6 months group. The level of microRNA-200c was significantly reduced in the PD > 6 months group, suggesting that microRNA-200c may be associated with EMT.

## Introduction

Peritoneal dialysis (PD) is the main treatment for patients with end-stage renal disease. However, peritoneal fibrosis (PF) caused by long-term PD eventually leads to ultrafiltration failure, which limits the application of PD (1,2). Human peritoneal mesothelial cells (HPMCs) constitute the largest cell population in the peritoneum. They maintain the peritoneal structure and stable function, preventing abdominal infection and peritoneal sclerosis. The epithelial-mesenchymal transition (EMT) is an important cause of PF in the PD process (3,4). The loss of epithelial cell adhesion and phenotypic transformation of fibroblasts to muscle are key steps in EMT (5).

Traditional HPMC isolation and culture is difficult to perform. In this study, the HPMCs shed from the peritoneal effluent of PD patients were separated and cultured. The HPMCs of PD patients reflect the function of peritoneal cells and may be analyzed for changes in the gene expression of cellular proteins E-cadherin, vimentin, fibronectin (FN) and COL-1. This is an effective method for investigating the physiological functions and changes in HPMCs in PD patients, and provides clinical evidence for reversing or preventing ultrafiltration failure.

A microRNA (miRNA) is a functional non-coding small molecule RNA existing in plant and animal genomes, with 21–25 nucleotides. miRNA binds with the untranslated region of the 3′ end, the coding region or the untranslated region of 5′ end of the target mRNA. This inhibits translation or triggers the degradation of mRNA and therefore inhibits translation following gene transcription. It has been demonstrated that miRNA directly regulates 30% of the transcription of protein-coding genes in the mammalian genome. miRNA plays an important role in cells and is involved in almost all biological processes (6,7). Previously, it has been shown that target miRNA may effectively reverse EMT and inhibit fibrosis of organs, including the heart, liver, lung and kidney. (8–11). It has also been shown that miRNA is a therapeutic target and a diagnostic marker (12–14). However, the role of miRNA in peritoneal mesothelial cell EMT and PF is unclear.

MicroRNA-200 (miRNA-200) is a recently discovered family that is closely associated with a variety of fibrotic diseases, and includes miR-200a, miR-200b, miR-200c, miR-141 and miR-429. The abnormal expression of the miRNA-200 gene cluster or a single gene has been observed in a variety of fibrotic diseases, including liver fibrosis, pulmonary fibrosis, kidney fibrosis and systemic sclerosis (15–17). Regulation of its expression may inhibit or reduce fibrosis, suggesting that miRNA-200 may be a potential anti-fibrotic target. In the present study, the changes in miR-200c expression levels in the HPMCs of PD patients were studied.

## Materials and methods

### Patients

The study included 28 patients with continuous ambulatory peritoneal dialysis (CAPD) without peritonitis, including 13 males and 15 females, aging from 41 to 73 (54.37±9.33) years old. There were 12 patients with newly inserted tubes (dialysis time ≤15 days; PD start group) and 16 patients who had been undergoing PD for >6 months (the PD >6 months group) with a dialysis time of 6–22 (10.80±4.38) months and a dialysate concentration of 2.5% (Baxter, Deerfield, IL, USA). There were 13 patients with chronic glomerulonephritis, 8 patients with diabetic nephropathy and 7 patients with hypertensive nephropathy. Prior written and informed consent were obtained from every patient and the study was approved by the ethics review board of Central South University (Changsha, China).

### Reagents

Fetal calf serum (15% FCS; Hangzhou Evergreen Biotechnology Co., Hangzhou, China) was added to DMEM/F12 medium (Gibco, Carlsbad, CA, USA). Goat E-cadherin antibody, mouse vimentin antibody, rat anti-human factor VIII antibody, leukocyte CD45, rabbit FN antibody, rabbit COL-1 antibody, mouse GAPDH antibody and goat anti-mouse horseradish peroxidase (HRP) labeled secondary antibody was purchased from Santa Cruz Biotechnology, Inc. (Santa Cruz, CA, USA). TRIzol reagent was purchased from Invitrogen (Carlsbad, CA, USA). The Revert Aid™ First strand cDNA Synthesis kit was purchased from Fermentus (Vilnius, Lithuania). SYBR GreenER™ qPCR SuperMix, the miRNeasy Mini kit and miRNA Q-PCR Detection kit were purchased from Invitrogen (USA).

### Isolation and culture of HPMCs

Samples of sterile abdominal effluent (~2,000 ml) were collected from the CAPD patients at night. The samples were centrifuged at 500 × g and 4°C for 5 min and the supernatant was discarded. The precipitated cells were washed twice with D-Hank’s balanced salt solution. Cells resuspended in 15% FCS DMEM/F12 medium were added to adjust the cell number to 1×10^6^ cells/ml and inoculated on gelatin-coated 25 mm^2^ (Corning, New York, NY, USA). The cells were cultured at 37°C in a 5% carbon dioxide incubator and the culture medium was changed every 72 h. The cultured cells were detected under an inverted phase contrast microscope (Eclipse TS100-F; Nikon, Tokyo, Japan) and by immunofluorescence and scanning electron microscopy (Philips CM 120 transmission electron microscope, Amsterdam, The Netherlands).

### Immunofluorescence

The cells were washed with PBS three times and fixed with 2–4% formaldehyde for 15 min. Non-immune goat serum containing 0.3% Triton X-100 was added and the cells were kept at room temperature for 60 min. The primary antibody was added at 4°C overnight. The primary antibodies used were anti-E-cadherin antibody, anti-Vimentin, anti-A cyclase VIII Antibody (B-6) and anti-CD45 antibody. The cells were washed three times with PBS for 5 min. The secondary antibody (50 *μ*l; 1:200) was added and the cells were kept at room temperature for 60 min. The secondary antibodies were Alexa Fluor^®^ 488 goat anti-mouse IgG2b (H+L) and Alexa Fluor^®^ 488 mouse anti-goat IgG (H+L). Finally, the cells were stained with DAPI. After washing three times with PBS, the cells were observed under a fluorescence microscope.

### Electron microscopy

The cells were washed three times with cold PBS and fixed with 2.5% glutaraldehyde for 30 min. After washing three times with PBS for 5 min, the cells were fixed with 1% osmium tetroxide for 4 h at 4°C. The cells were dehydrated with ethanol. After drying and gilding by ion sputter, the cells were detected by scanning electron microscopy.

### qPCR

Total cell RNA was extracted with TRIzol reagent. The cDNA synthesis was performed according to the manufacturer’s instructions for the RevertAid™ H Minus FirstStrand cDNA Synthesis kit. The PCR amplification was performed using the SYBR GreenER qPCR SuperMix according to the manufacturer’s instructions. Deionized water was the negative control for each PCR. The relative expression amount of target gene mRNA=2^−ΔΔCt^. The Ct value was the cycle number before fluorescence reached the threshold value. ΔΔCt=(Ct target gene - Ct housekeeper gene) experimental group - (Ct target gene - Ct housekeeper gene) control group. In this experiment, the PD start group was considered the control group. The relative expression level of mRNA in the more than 6 months PD group was expressed as 2-ΔΔCt in the PD start group. The primers used in this study are shown in Table I.

### Western blotting

Total protein was extracted for 12% SDS-PAGE electrophoresis. The primary antibodies against E-cadherin (1:500), vimentin (1:800), FN (1:500) and COL-1 (1:500) were used and incubated at room temperature for 1 h. Secondary antibodies used in this study were goat anti-mouse IgG-HRP (Cat# sc-2005, 1:10,000; Santa Cruz Biotechnology, Inc.). After development by enhanced chemiluminescence (ECL), gel pro 4.0 software (Media Cybernetics, Rockville, MD, USA) was used for image analysis.

### Statistical analysis

All data were analyzed by SPSS 11.0 statistical software (SPSS, Inc., Chicago, IL, USA). The experimental data are expressed as mean ± standard deviation. A Student’s t-test was used to compare values. P<0.05 was considered to indicate a statistically significant result.

## Results

### Morphological changes of HPMCs in patients

**Figure 1.**
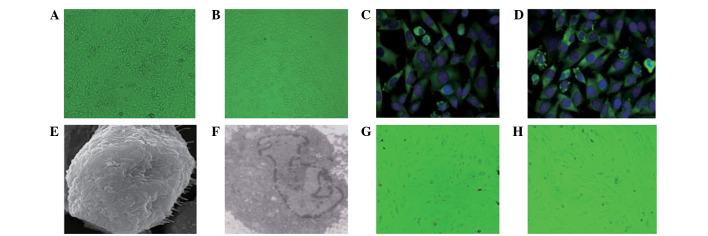
Observation of HPMCs. Observation of HPMCs under a microscope at (A) day 2 after primary culture (magnification, ×200; observed under light microscope, no staining) and (B) day 14 after primary culture (magnification, ×200; observed under light microscope, no staining). Immunofluorescence showed (C) vimentin-positive HPMCs (magnification, ×400; immunofluorescence staining) and (D) cytokeratin-positive HPMCs (magnification, ×400; immunofluorescence staining). (E) Electron microscopy showed an HPMC in PD effluent culture (magnification, ×10,000; gilded with ion sputter). (F) Transmission electron microscopy of an HPMC in PD effluent culture (magnification, ×4,000; gilded with ion sputter). (G) Mixed mesothelium (magnification, ×200; observed under light microscope, no staining). (H) Fibroblast-like mesothelium (magnification, ×200; observed under light microscope, no staining). (A–G) HPMCs were from the PD start group; (H) HPMCs were from the PD >6 months group. HPMCs, human peritoneal mesothelial cells; PD, peritoneal dialysis.

To determine the morphological changes in the HPMCs of patients, the cells were detected using microscopy, immunofluorescence labeling, electron microscopy and transmission electron microscopy. The results of the PD start group is shown in Fig. 1A–G and the results of the PD >6 months is shown in Fig. H. Effluent HPMCs were fusiform after 2 days of primary culture and were not easily differentiated from fibroblasts (Fig. 1A). At 14 days, cells were fused (Fig. 1B). In order to determine the sources of the HPMCs, immunofluorescence detection was performed. This showed that cytoplasmic vimentin (Fig. 1C) and cytokeratin (Fig. 1D) antigens were positively detected in the cells. A visible green fluorescent area in the cytoplasm was considered a positive result. The cell nucleus was restained with DAPI in order to show the complete nucleus (Fig. 1C and D). There was no fluorescent signal for factor VIII and leukocyte CD45, suggesting that there was no factor VIII and leukocyte CD45 expression in the cell and the cell purity was >95%. Scanning electron microscopy showed filamentous microvilli of different lengths on the cell surface (Fig. 1E). Transmission electron microscopy showed a large number of microvilli on the cell surface and abundant endoplasmic reticulum and mitochondria in the cytoplasm without Weibel-Palade bodies (Fig. 1F).

Through a light microscope, the cells in the PD effluent were observed to be round or oval epithelioid cells, spindle fiber-like cells or mixed HPMCs. Certain HPMCs of the PD start group were round or oval-shaped epithelioid cells. After fusion, cells were cobblestone-like, similar to omentum-cultured HPMCs. However, the majority of epithelioid cells and spindle cells were mixed (Fig. 1G). Spindle fiber-like cells were mainly observed in patients who had been undergoing PD for >6 months (Fig. 1H). The cell morphological changes indicate that the cells from the peritoneal dialysate effluent fluids are HPMCs, which suggests the occurrence of EMT.

### E-cadherin, vimentin, FN and COL-1 expression of peritoneal mesothelial cells in PD patients

**Figure 2.**
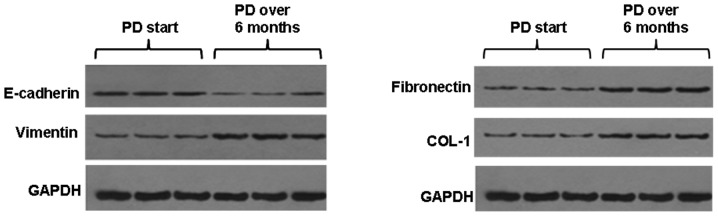
Western blotting. Differences in the expression of E-cadherin, vimentin, fibronectin and COL-1 protein in the PD start and PD >6 months groups. Total protein was extracted for western blot analysis. The primary antibodies against E-cadherin (1:500), vimentin (1:800), fibronectin (1:500) and COL-1 (1:500) were used. Secondary antibodies used in this study were goat anti-mouse IgG-HRP. After development by ECL, gel pro 4.0 software was used for image analysis. Blot results from three patients are shown. PD, peritoneal dialysis.

To determine the expression levels of proteins, HPMCs were harvested from the patients in the PD start group and the PD >6 months group. The western blot analysis showed that E-cadherin protein expression levels in the peritoneal mesothelial cells were significantly reduced in the PD >6 months group (P<0.05) and the vimentin, FN and COL-1 protein expression levels were significantly increased (P<0.05) compared with those in patients in the PD start group (Fig. 2).

**Figure 3.**
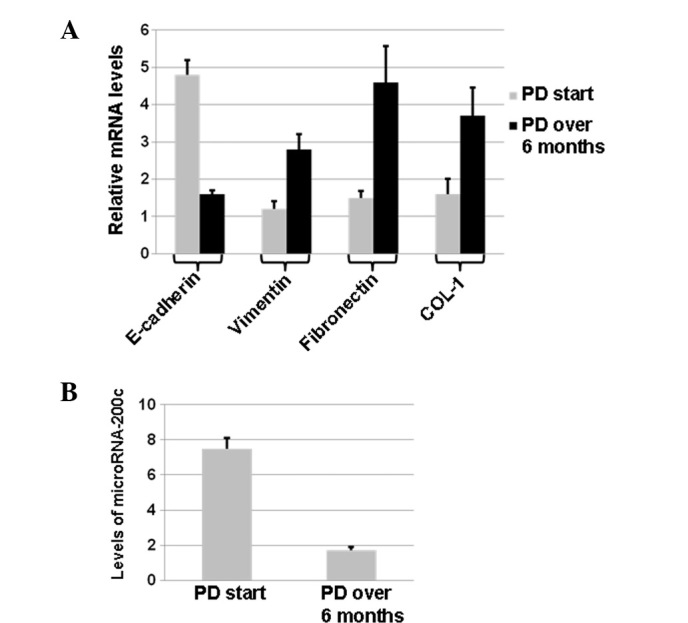
qPCR. (A) Differences in the expression of E-cadherin, vimentin, fibronectin and COL-1 mRNA in the PD start and PD >6 months groups. (B) miRNA-200c expression in the PD start and PD >6 months groups. U6 served as the internal reference of qPCR. PD, peritoneal dialysis.

qPCR also showed that E-cadherin mRNA levels in the peritoneal mesothelial cells were significantly decreased in the PD >6 months group (P<0.05), and vimentin, COL-1 and FN mRNA levels were significantly increased (P<0.05) (Fig. 3A). These results suggest that the expression of these molecular marker proteins was altered in different ways at the transcriptional levels.

### miRNA-200c expression changes in the effluent peritoneal mesothelial cells of PD patients

To determine the changes in miRNA-200c expression, qPCR was performed. With U6 as a reference gene, miRNA-200c expression in the peritoneal mesothelial cells of PD patients was determined by qPCR Taqman probe assay. The results showed that the relative expression of miRNA-200C was 7.546±0.341 in the PD start group and 1.701±1.070 in the PD >6 months group; the expression level was significantly lower in the PD >6 months group than in the PD start group (P<0.05; Fig. 3B). These results suggest that changes in miRNA-200c levels may be associated with EMT.

## Discussion

EMT is an important source of formation of myofibroblasts (18,19). In 2003, morphological changes in the HPMCs of PD effluents were first reported, including in the epithelial cells and the fiber cells (20). In the present study, HPMCs were separated and cultured from the PD effluent with a cell purity of >95%. At one and two days after primary culture, the effluent HPMCs were fusiform and could not be differentiated from fibroblasts until 10-4 days. Immunofluorescence and scanning electron microscopy analysis of the effluent HPMCs was positive for vimentin, cytokeratin and microvilli on the cell surface. By contrast, myofibroblasts were positive for vimentin and negative for cytokeratin on the cell surface. This indicates that the myofibroblasts fiber-like cells were derived from mesothelial cells, which indicated the occurrence of EMT.

Previous *in vivo* experiments have indicated that are were fiber-like cells in the peritoneal tissues of long-term PD patients, which not only express typical epithelial cell markers but also expressed fibroblast markers, such as α-SMA (21). It has also been demonstrated that EMT-related changes in peritoneal mesothelial cells may present in early peritoneal dialysis (20). As the time of PD is extended, epithelial cells gradually change to a shuttle type and the expression of epithelial cell marker E-cadherin decreases, while the expression of mesenchymal marker vimentin increases (22,23). Previously it was shown that in PD patients with 2 years of dialysis, 74% lost mesothelial cells in the peritoneum, 46% had PF and 17% showed evidence of in situ EMT in the peritoneum (24). There were myofibroblasts in all peritonea with EMT changes. In this experiment, it was observed that in the PD start group, the mesothelial cells in the peritoneal dialysis effluent were round, oval and mixed epithelioid cells. For the mixed cells, EMT of the peritoneal mesothelial cells was detected in early peritoneal dialysis. However, as the dialysis time was extended, the cell morphology changed significantly, and the cells in the PD more than 6 months group were long spindle fiber-like. We observed that the expression level of E-cadherin was decreased, whereas the expression of vimentin, Col-1 and FN was significantly increased in the PD >6 months group, suggesting that the protein expression was changed significantly. In the early stage of CAPD, when cells remain cubic, the expression levels of E-cadherin, vimentin, FN and COL-1 changed, indicating EMT is the starting point of PF. When the peritoneum is exposed to a mechanical exfoliation environment for a long time, the phenotype inevitably becomes abnormal and mesothelial cells are completely transformed due to injury. This promotes fibrosis and ultrafiltration failure. These molecular protein markers provide evidence for further study of the molecular mechanisms of PF. We also observed that the level of microRNA-200c was significantly reduced in the PD >6 months group. This finding suggests that microRNA-200c may be involved in the EMT process.

## Figures and Tables

**Table I.** The primers used in this study.

Primers	Sequences	Product size (bp)
E-cadherin forward	5′-TCATGAGTGTCCCCCGGTAT	240
E-cadherin reverse	5′-TCTTGAAGCGATTGCCCCAT	
Vimentin forward	5′-GCTACGTGACTACGTCCACC	265
Vimentin reverse	5′-TAGTTGGCGAAGCGGTCATT	
FN forward	5′-AACTGGTAACCCTTCCACACCC	266
FN reverse	5′-AGCTTCTTGTCCTACATTCGGC	
COL-1 forward	5′-GCCAAGACGAAGACATCCCA	156
COL-1 reverse	5′-GGCAGTTCTTGGTCTCGTCA	
GAPDH forward	5′-CAATGACCCCTTCATTGACC	106
GAPDH reverse	5′-GACAAGCTTCCCGTTCTCAG	
hsa-miR-200c forward	5′-TAATACTGCCGGGTAATGATGGA	75
hsa-miR-200c reverse	5′-TGGTGTCGTGGAGTCG	
U6 forward	5′-GCTTCGGCAGCACATATACTAAAAT	81
U6 reverse	5′-CGCTTCACGAATTTGCGTGTCAT	

FN, fibronectin.
